# Adverse Events Following Vitreoretinal Surgeries Under Adequacy of Anesthesia with Combined Paracetamol/Metamizole—Additional Report

**DOI:** 10.3390/jcm14176261

**Published:** 2025-09-04

**Authors:** Kaja Marczak, Michał J. Stasiowski, Anita Lyssek-Boroń, Nikola Zmarzły

**Affiliations:** 1 Department of Anaesthesiology and Intensive Care, 5th Regional Hospital, 41-200 Sosnowiec, Poland; kaja.marczak.wss5@gmail.com; 2 Chair and Department of Emergency Medicine, Faculty of Medical Sciences in Zabrze, Medical University of Silesia, 40-055 Katowice, Poland; 3 Department of Ophthalmology with Paediatric Unit, 5th Regional Hospital, Trauma Centre, 41-200 Sosnowiec, Poland; anitaboron3@gmail.com; 4 Department of Ophthalmology, Faculty of Medicine, Academy of Silesia, 40-055 Katowice, Poland; 5 Collegium Medicum, WSB University, 41-300 Dabrowa Gornicza, Poland; nikola.zmarzly@wsb.edu.pl

**Keywords:** vitreoretinal surgery, general anesthesia, paracetamol, metamizole, postoperative nausea and vomiting, Adequacy of Anesthesia, oculocardiac reflex, oculoemetic reflex, numeric pain rating scale, intravenous preventive analgesia

## Abstract

**Background/Objectives**: Some patients undergoing vitreoretinal surgery (VRS) require general anesthesia (GA), despite the possibility of developing intolerable postoperative pain perception (IPPP). Intraoperative rescue opioid analgesia (IROA) administration during GA poses a risk of perioperative nausea and vomiting (PONV), which may result in suprachoroidal hemorrhage with permanent visual impairment. Adequacy of Anesthesia (AoA) optimizes intraoperative IROA titration. Intravenous preemptive analgesia (IPA) with cyclooxygenase-3 (COX-3) inhibitors is added to GA to reduce the IROA dose. In this additional analysis, we assessed the impact of preemptive analgesia with COX-3 inhibitors, administered alongside GA with AoA-guided IROA, on the incidence of PONV, oculocardiac reflex (OCR), and oculoemetic reflex (OER) in patients undergoing VRS as secondary outcomes. **Methods**: A total of 165 patients scheduled for VRS were randomly assigned to receive AoA-guided GA combined with IPA at a single dose of 1 g of paracetamol (acetaminophen) or 2.5 g of metamizole or both. A total of nine patients were excluded due to technical problems with the intraoperative surgical pleth index (SPI) measurement, inability to report postoperative pain, and postoperative arousal resulting in a loss of follow-up in Stage 5. **Results**: Regardless of the group assignment, AoA guidance of GA resulted in PONV in 4%, OCR in 10%, and OER in 0% of the 153 analyzed patients undergoing VRS. No significant differences were observed between the groups regarding the type of IPA. PONV was observed in 2.11% (3/142) of patients with zero, one, or two risk factors of PONV, as compared to 27% (3/11) of patients with at least three PONV risk factors, assessed using the Apfel score. **Conclusions**: IPA with both paracetamol and metamizole did not demonstrate a benefit in reducing the analyzed adverse events compared with their single use in patients undergoing VRS under AoA guidance during GA. Surprisingly, PONV was hardly observed in patients with zero, one, or two PONV risk factors assessed by the Apfel score who underwent AoA-guided VRS during GA with IPA using one or two COX-3 inhibitors.

## 1. Introduction

Postoperative nausea and vomiting (PONV) constitutes one of the most serious complications following vitreoretinal surgery (VRS) and may lead to suprachoroidal hemorrhage, regardless of the use of regional anesthesia (RA) with monitored anesthesia care (MAC) [1] or general anesthesia (GA) due to contraindications for RA alone [2,3]. Rupture of the posterior ciliary arteries or vortex veins after PONV can result in suprachoroidal hemorrhage, leading to permanent visual impairment, with an incidence of up to 1% after VRS [4].

Strategies to reduce the adverse events of PONV include pharmacological approaches, such as antiemetic prophylaxis [5], opioid-free anesthesia [6], premedication with benzodiazepines [7,8] or anxiolytics [9], and the use of a muscle relaxant antagonist for smooth recovery from GA [10], as well as non-pharmacological interventions, including aromatherapy [11], chewing gum [12], cheek acupuncture [13], or the intraoperative use of noise-canceling headphones [14]. RA has been shown to reduce the cumulative dose of intravenous rescue opioid analgesia (IROA), an independent risk factor for PONV after VRS [15]. The employment of different preoperative RA techniques or intravenous preventive analgesia (IPA) added to GA in patients undergoing VRS decreases IROA requirements [16,17,18], enhances the effectiveness of postoperative analgesia [19], and reduces the incidence of PONV and oculocardiac reflex (OCR) [16].

Surgical maneuvers under GA induce painful afferent stimulation (nociception). Insufficient intraoperative analgesia (anti-nociception) leads to the release of stress hormones, which increases heart rate and arterial blood pressure, while IROA administration attenuates these effects. Underdosing IROA during GA may evoke intolerable postoperative pain perception (IPPP) [20] due to central sensitization [21], whereas overdosing may result in hemodynamic instability, including bradycardia and hypotension. Volatile anesthetics can impair hemodynamic responses to painful stimuli, particularly in patients with diabetic and elderly patients [22], often requiring VRS under GA. Consequently, the use of digital devices for the intraoperative monitoring of nociception/anti-nociception balance is steadily increasing [23].

Adequacy of Anesthesia (AoA) guidance for GA facilitates intraoperative monitoring [24]. The surgical pleth index (SPI; 0–100) reflects nociceptive stimulation and recovery after affective anti-nociception. Response entropy (RE) and state entropy (SE), ranging from 45 to 60, indicate the depth of surgical anesthesia. The SPI signal is derived from finger photoplethysmography, while SE and RE signals are derived from a forehead sensor, eliminating time-consuming preparation prior to GA induction [25]. The SPI correlates with IROA serum concentrations [26] and allows a more reliable titration than hemodynamic monitoring alone [27,28,29], reducing cumulative IROA during GA [30] and the IPPP incidence assessed by the numeric pain rating scale (NPRS) [31]. The SPI guidance in terms of the nociception/anti-nociception balance, together with the depth of anesthesia monitored with the bispectral index, has been reported to halve the PONV incidence after thoracoscopic lung resection, albeit with a longer stay in the postoperative care unit (PACU) [32].

In our previous study, preventive analgesia did not affect the overall IPPP incidence in patients undergoing VRS when IROA was titrated under the SPI guidance, suggesting that AoA guidance attenuates the effect of preventive analgesia [33]. However, we also observed reduced PONV incidence in patients receiving IPA compared with preventive RA, topical anesthesia, and the control group, with decreased IROA requirements in patients receiving paracetamol and peribulbar block compared with metamizole, topical anesthesia, and the control group [34].

To date, no study has investigated the effect of single-dose IPA with paracetamol (1 g), metamizole (2.5 g), or their combination on the adverse event incidence in patients undergoing VRS under AoA guidance during GA, in line with the recent guidelines for postoperative pain management after head and neck surgery [35].

A previous analysis of primary outcomes showed a reduced IPPP incidence without affecting hemodynamic stability in patients receiving both cyclooxygenase-3 (COX-3) inhibitors compared to metamizole alone, and lower IROA requirements in the paracetamol group compared to the metamizole group [36]. Based on these findings and the current literature [37], we analyzed secondary outcomes to evaluate the effect of combined or single IPA on OCR, PONV, and oculoemetic reflex (OER) in patients undergoing VRS under AoA guidance.

## 2. Materials and Methods

Patients scheduled for VRS at the Department of Ophthalmology (DO) of the 5th Regional Hospital in Sosnowiec, who met the inclusion criteria, were invited to participate in this study. After obtaining written informed consent, 165 patients classified as ASA I-III according to the American Society of Anaesthesiologists were enrolled. Patients were then randomly assigned to groups using the sealed envelopes method.

In compliance with the Declaration of Helsinki, the Bioethical Committee of Medical University of Silesia (Chairman Ph. Dr. B. Okopień) issued approval for this study (KNW/0022/KB1/122/17; 5 December 2017). This study was recorded in the Clinical Trial Registry (Silesian MUKOAiIT7, NCT03389243; 27 December 2017), and data were collected from 15 January 2018 to 16 December 2022.

Exclusion criteria included the following: acute or chronic pain, drug or alcohol abuse, pregnancy, history of pulmonary disease, neurological disorders or prior neurosurgical procedures that could interfere with EEG entropy monitoring, allergy or abuse of metamizole or paracetamol, liver dysfunction, predictors of difficult laryngeal mask placement, and cardiac arrhythmia on ECG that could complicate the SPI monitoring.

Patients were randomly assigned to three groups. In the P group, AoA-guided GA combined with single-dose IPA of 1 g of paracetamol in 100 mL saline (Paracetamol Kabi 10 mg/mL; Fresenius Kabi, Błonie, Poland) was administered 30 min before arrival in the operating room. In the M group, AoA-guided GA combined with a single-dose IPA of 2.5 g of metamizole in 100 mL saline (Pyralgin 0.5 g/mL; Polpharma S.A., Gdansk, Poland) was administered 30 min before arrival in the operating room. In the PM group, patients received AoA-guided GA combined with both IPA interventions as described for the P and M groups, in accordance with recent postoperative pain management guidelines [35].

During the preoperative visit, patients were educated on postoperative pain and trained to report pain using the NPRS (zero corresponded to no pain and 10 to the worst pain they could imagine).

All patients fasted for at least 12 h. On the day of surgery, patients received age- and weight-adjusted dose of midazolam (3.75–7.5 mg/kg; Dormicum, Roche, Warsaw, Poland) prior to the induction of anesthesia [38]. Patients were preoxygenated with 100% oxygen for 5 min and received intravenous Ringer’s Solution at 10 mL/kg. GA was induced intravenously with fentanyl (FNT) at 1 mcg/kg and a single dose of 2 mg/kg of propofol (Etomidate Lipuro, Braun, Germany). After loss of consciousness, 0.6 mg/kg of rocuronium (Esmeron, Fresenius, Błonie, Poland) was administered intravenously to paralyze patients, and after 45 s, a laryngeal mask was placed. CO2 was maintained at 35–37 mmHg. After laryngeal mask installation, before VRS started, the sevoflurane concentration was maintained at approximately 40–45 SE. Standard monitoring included the monitoring of non-invasive blood pressure (NIBP), heart rate (HR), standard electrocardiography (ECG) II, arterial oxygen saturation (SaO2), fraction of inspired oxygen (FiO2), end-tidal carbon dioxide (etCO2), minimum alveolar concentration of sevoflurane, and fraction of inspired and expired sevoflurane (FiAA and FeAA).

Entropy EEG (SE, RE) was used to monitor anesthesia depth, SPI guided the management of intraoperative analgesia, and muscle relaxation was ensured via neuromuscular transmission monitoring (Carescape B650, GE Healthcare, Helsinki, Finland).

### 2.1. Stage 1

On arrival in the operating room, patients were fitted with EEG entropy sensors (SE, RE) on their forehead, the SPI sensors on their finger opposite to the venous access, and an NIBP cuff on their right arm, while standard ECG electrodes were placed on the patients’ backs. First values were then recorded.

### 2.2. Stage 2

The mean SPI value was calculated from 5 min following laryngeal mask placement up to the start of orbital sterilization, allowing for sensor calibration.

### 2.3. Stage 3—Intraoperative Analgesia

The SPI was recorded every minute. A rescue dose of 1 mcg/kg of FNT was administered intravenously every 5 min when ∆SPI exceeded 15 points above the Stage 2 baseline until the SPI returned to the baseline. VRS duration was measured from the installation of the speculum to its removal.

We assumed that an initial FNT dose of 1 mcg/kg would provide sufficient analgesia before speculum installation. Furthermore, Gruenewald et al. [23] suggested that insufficient analgesia could be predicted by either a ∆SPI greater than 10 or an absolute SPI value above 50. In some studies, only an absolute ∆SPI value > 50 has been used as an indication for rescue analgesia [39]. To account for SPI variability and avoid a potentially dangerous overdose of FNT, a ∆SPI > 15 compared with the Stage 2 baseline, maintained for a minimum of one minute, served as an indicator of rescue analgesia. All surgeries were conducted by the ophthalmic surgeon (A. L-B) with >10 years of experience and >400 procedures annually.

The incidence of intraoperative OCR, which typically occurs when HR decreases by 20% during ocular manipulation, was recorded. When OCR was observed, stimulation in the surgical field was temporarily halted, and a single intravenous dose of 0.5 mg of atropine was administered if HR did not normalize. Persisting hypotension was managed with 5 mL/kg of crystalloid. A single dose of 5 mL of ephedrine (Ephedrinum hydrochloricum WZF 25 mg/mL, Polfa Warszawa S.A., Warsaw, Poland) was administered if the infusion did not raise the mean arterial pressure (MAP) above 65 mmHg.

### 2.4. Stage 4—Emergence from GA

During recovery from GA, the anesthesia team continued to monitor patients for their SPI, HR, SAP, MAP, DAP, and SaO2.

### 2.5. Stage 5—Postoperative Monitoring

In the PACU, patients were further monitored (SPI, HR, SAP, MAP, DAP, SaO2) by the anesthesiology team, blinded to the patient group assignment. In addition to postoperative hemodynamic parameters, each patient was monitored for adverse effects such as nausea, vomiting (PONV), allergic reactions, and sedation level, along with pain assessment for 24 h. For the management of PONV, patients received 4 mg of ondansetron (Ondansetron Accord, Accord Healthcare Limited, Harrow, UK) intravenously. If ondansetron was ineffective, a single dose of 4 mg of dexamethasone (Dexaven, Jelfa, Jelenia Góra, Poland) was administered intravenously. A 5 mL/kg quantity of Optilyte solution was infused when the MAP was <65 mmHg. Patients were provided with oxygen at 3 L/min through a nasal cannula. Patients were queried about their perception of pain intensity using the NPRS every 10 min. For an NPRS of >3, a standard dose of non-steroidal anti-inflammatory drugs (NSAIDs), such as paracetamol or metamizole, was provided, according to the current guidelines for acute pain management issued by the Polish Society of Anaesthesiologists [20]. The SPI was recorded every minute. The NPRS and SPI were recorded for mild pain (NPRS 0–3), average pain (NPRS 4–6), and acute pain (NPRS 7–10). Patients were monitored in the PACU for at least 30 min until transfer to the DO. Monitoring and data recording were discontinued, except for the PONV incidence, which was recorded until discharge from the DO.

The Apfel scores were calculated preoperatively to ensure group homogeneity. PONV risk factors include female gender, history of motion sickness or PONV, non-smoking status, and postoperative opioid administration. The estimated PONV incidence was 10%, 21%, 39%, 61%, and 79% corresponding to the presence of zero, one, two, three, or four risk factors, respectively [40,41]. PONV was categorized as early when it occurred in the PACU and late when it occurred in the DO. Overall PONV was defined as the presence of either early or late PONV, or both.

### 2.6. Statistical Analysis

Statistical analyses were conducted with STATISTICA 13.3 (StatSoft, Kraków, Poland) and R (ver. 4.4.0) [42]. Continuous data were presented as mean with standard deviation (X ± SD) and median (Me) with interquartile range (IQR). First, normality was determined with the Shapiro–Wilk test. The Kruskal–Wallis test, followed by Dunn’s post hoc test, was then employed to compare continuous variables across groups. Nominal variables were reported as percentages and compared between groups using the Chi-square test, or Fisher’s exact test in cases where any expected frequency was under 5. Bonferroni correction was applied for multiple testing. To account for potential imbalances between groups in gender, the BMI, and the Apfel score, multivariable logistic regression was conducted. Odds ratios (ORs) with 95% confidence intervals (CIs) were calculated for each predictor. Post hoc pairwise comparisons between all three treatment groups were performed using estimated marginal means with Tukey adjustment for multiple comparisons. Statistical significance was set at *p* < 0.05.

G*Power (ver. 3.1.9.7; Heinrich Heine University, Düsseldorf, Germany) was used to calculate group size. One-way ANOVA (f = 0.25, α = 0.05, power = 0.8) for 3 groups determined the total sample size of 159. As data from 153 patients was analyzed, a post hoc test showed a power of 0.79.

## 3. Results

**Figure 1:**
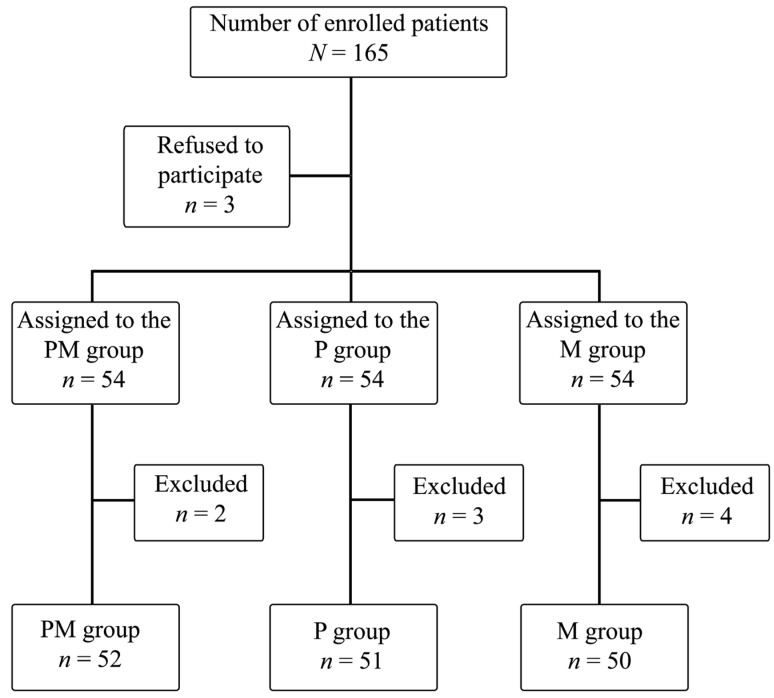
Randomization scheme for study participants.

A total of nine patients were excluded from this study due to technical problems with the intraoperative SPI measurement, inability to report postoperative pain, and postoperative arousal resulting in loss of follow-up in Stage 5. The final analysis included 153 patients (Figure 1).

Significant differences between groups were observed in height, weight, the BMI, and HA. The PM group had a significantly higher proportion of men compared to the P group (Table 1).

Intraoperative FNT consumption was significantly higher in the M group than in the P group (Table 2).

Although the PM group had a significantly higher Apfel score (%) than the P group, no significant differences were found in their patients’ medical histories (Table 3).

Postoperative pain intensity, assessed using the NPRS, did not differ significantly between groups. Mild pain was more frequently reported in the PM group compared to the M group, while acute pain was observed only in the PM and P groups (Table 4).

The IPPP incidence was higher in the M group compared to the PM group. The incidence of PONV and OCR was not affected by the use of various preemptive analgesia methods (Table 5).

In addition, multivariable logistic regression analyses with Tukey-adjusted estimated marginal means were performed to evaluate the incidence of overall PONV and OCR while adjusting for potential confounders, including gender, BMI, and the Apfel score (%), accounting for observed group imbalances. For PONV, no statistically significant differences were observed between any groups (M vs. MP OR = 0.17, 95% CI: 0.009–3.21, *p* = 0.33; M vs. P OR = 0.86, 95% CI: 0.03–24.68, *p* = 0.99; MP vs. P OR = 5.08, 95% CI: 0.27–95.93, *p* = 0.40). Similarly, for OCR, no significant differences were observed: M vs. MP OR = 1.75 (95% CI: 0.29–10.41, *p* = 0.74), M vs. P OR = 1.19 (95% CI: 0.28–5.19, *p* = 0.96), MP vs. P OR = 0.68 (95% CI: 0.11–4.10, *p* = 0.87). The Apfel score, the BMI, and gender did not significantly influence the incidence of PONV or OCR. The wide confidence intervals, particularly for PONV, reflect the low number of events and increased uncertainty around the estimated odds ratios.

Further analysis examined the correlation between the overall PONV incidence and the number of PONV risk factors according to the Apfel score. Significantly more patients were at risk due to the presence of one or two separate risk factors than zero or three risk factors. Despite this, no statistically significant difference was observed in the overall PONV incidence based on the number of V risk factors. There were no patients with an Apfel score of 4, as opioid analgesics were not required to alleviate postoperative pain (Table 6).

## 4. Discussion

The growing prevalence of diabetic and geriatric populations has increased the requirements for VRS, while comorbidities, such as diabetes, severe hepatic insufficiency, the chronic dysfunction of the respiratory, hepatic, renal, or other major organs, antiplatelet therapy, and the risk of globe perforation due to impaired patient cooperation during prolonged procedures caused by psychotic illness, depression, chronic pain, or other disabilities, may potentially preclude VRS under RA alone with MAC [1]. Consequently, the necessity for GA regimens involving IROA encouraged the exploration of IPAs, which have been reported to reduce IROA requirements and, in turn, decrease the incidence of perioperative adverse events such as PONV, OCR, IPPP, similar to RA techniques used adjunctively with GA for preventive analgesia [36].

Paracetamol (acetaminophen) and metamizole are among the most widely used non-opioid analgesics, exerting both central (COX-3 inhibition) and peripheral effects [43,44,45,46,47]. Paracetamol has been reported to act via cannabinoid receptor CB1, activating internal cannabinoids [48], potentially contributing to the sparing effects on IROA [49]. Metamizole acts through the PI3Kγ/AKT signaling cascade, a well-established molecular mechanism promoting nitric oxide production in sensory neurons [50]. Their analgesic efficacy in managing IPPP, even when administered as a single IPA dose, has been widely reported [51,52,53,54,55,56]. These agents also reduce the PONV incidence [52,54,57], and their relatively good safety profile is consistently emphasized [53,55,56,58]. Therefore, their use is preferred over standard NSAIDs, such as ketorolac, particularly in patients with upper gastrointestinal and kidney diseases that increase the risk of NSAID-related complications [53,59,60].

In this study, despite the group allocation, an overall PONV rate of 4% (in 6 out of 153 patients) was observed, which is markedly lower than that reported in the current literature. In the study by Eberhart et al. [61], a standardized GA was performed, including premedication with benzodiazepine, propofol, atracurium, or vecuronium for GA induction and desflurane in N(2)O/O(2) with a continuous remifentanil infusion for GA in patients undergoing VRS (standard three-port pars plana vitrectomy for proliferative diabetic vitreoretinopathy, complicated retinal detachment, or macular disease). The reported PONV incidence was 56% (placebo), 40% (dolasetron), 28% (droperidol), and 18% (dolasetron and droperidol).

Similarly, low PONV rates after VRS, comparable to our findings, were reported by Reibaldi et al. [1] in awake patients receiving both ondansetron and dexamethasone for PONV prophylaxis prior to VRS under peribulbar block using ropivacaine with MAC and without IROA. Double prophylaxis resulted in no PONV in 95.96% of the patients, whereas dexamethasone alone prevented PONV in 80.79%, ondansetron alone in 80.38%, and placebo in 71.96% of the patients. Notably, 186 of 1937 patients (about 9.6%) were excluded due to diabetes, whereas patients with diabetes in whom fasting-related nausea is a recognized effect of diabetic gastroparesis [62] were included in this study. Our previous work also identified diabetes as an independent PONV risk factor in patients undergoing AoA-guided GA for VRS [63]. Consequently, AoA-guided GA with IPA using COX-3 inhibitors may represent the anesthetic regimen of choice for patients with diabetes excluded from VRS under RA with MAC.

Similarly to diabetes, underhydration with preoperative morning fasting have been reported to increase the PONV incidence via enhanced serotonin secretion [64]. Thus, intraoperative intravenous crystalloid administration may serve as an important preventive measure against PONV, representing a low-cost, non-pharmacological option to reduce its incidence, free from the side effects of pharmacological therapy [40]. In this study, no statistically significant differences in intraoperative fluid requirements were found between groups. We hypothesize that AoA guidance resulted in an indirectly comparable demand for intraoperative fluid administration due to a directly comparable requirement for IROA, as well as for intravenous and volatile anesthetics for the co-induction of GA; all of these factors proportionally impaired the systemic vascular resistance, creating a demand for intravenous crystalloids, while avoiding overhydration, which is a separate PONV risk factor. Overall, balanced mesenteric perfusion, which prevents gut ischemia or edema due to overhydration, may further reduce serotonin-mediated PONV [64].

Numerous anesthetic regimens have long been employed to reduce the need for IROA and, consequently, the PONV incidence, including the use of IPA to decrease IPPP and, thus, possibly reduce PONV. In cases where GA is required for VRS, and concomitant RA contraindications have a common limiting effect on the IROA dosage [16], an IPA regimen with the potential to achieve a similar effect is sought as a reasonable alternative to antiemetics. Both metamizole [52] and paracetamol [54] have demonstrated the aforementioned antiemetic potency.

In this study, PONV occurred in one patient assigned to the P group (2%), two patients assigned to the M group (4%), and three patients assigned to the MP group (6%), with no significant differences between the groups. This contrasts with the observation of a previous project, which formed the basis of this study, where IPA using either of the two COX-3 inhibitors, metamizole and paracetamol, reduced the PONV incidence compared to the regional techniques and control group, despite no preventive analgesic regimen reducing the IPPP incidence [34].

In one of the first studies employing AoA guidance, Bergmann et al. [30] observed a reduction in the cumulative IROA requirements during the SPI-guided GA. Consequently, our initial assumption that the combined limiting effect of IPA and AoA guidance on the IROA requirement would synergistically reduce both the IROA demand and PONV incidence was not confirmed. To sum up, IPA using either a paracetamol/metamizole combination or metamizole alone reduced the IPPP incidence but did not reduce PONV to a degree lower than that achieved with COX-3 inhibitors alone in the previous project under AoA guidance.

Mandelcorn et al. [15] observed postoperative nausea occurring almost three times more frequently in patients undergoing VRS under RA with MAC. Their patients required IROA due to incomplete RA block compared with those who did not need it due to a complete block. The authors determined that the use of IROA was the only variable associated with postoperative nausea. In this study, IPA with metamizole or paracetamol or their combination as an anesthetic modality led to a statistically significant difference in the cumulative IROA dose between the M and P groups, although it did not result in a proportional reduction in the PONV rate in the M group. Similar observations were made by Ohnesorge et al. [65] in their study on breast tumor surgery and by Sener et al. [66] in their study on tonsillectomy in children.

Regardless of these findings, the PONV incidence has been shown to be influenced by a number of other factors, including type of surgery, gender, smoking status, motion sickness, body weight, and history of PONV [40]. Its estimated incidence is approximately 30%, with nausea occurring in approximately 50% of patients, and in high-risk patients, the incidence of PONV may reach up to 80% [41,67,68,69]. Nitahara et al. [70] reported one or more episodes of PONV in 24% of adult patients undergoing VRS. Female gender, a lower BMI, and GA with inhalational anesthetics, also used in this study, were significantly associated with nausea within the first 2 h after VRS, whereas smoking did not influence the risk of PONV. Intravenous opioid analgesia, especially continuous oxycodone infusion, may elevate nausea incidence to 94% and vomiting to 26% [71]. Therefore, any IPA strategy aiming to reduce or avoid IROA is valuable.

According to the latest guidelines, standard prophylaxis with two antiemetics, such as dexamethasone in combination with a 5-HT3 receptor antagonist, should be the basis for treating PONV in every adult patient. In high-risk patients, the standard prophylaxis regimen should include a third and potentially a fourth antiemetic with a different mechanism of action [68]. The recent consensus guidelines advocate for assessing risk factors such as female gender, prior PONV or motion sickness, non-smoking status, postoperative opioid administration, younger age, surgery type, prolonged anesthesia, and volatile anesthetics, as well as reducing the patient’s baseline risk (e.g., via RA or employing non-opioid analgesics as a component of a multimodal strategy).

In this study, patients were assessed for the risk of PONV using the most popular method, i.e., the Apfel score. Among patients with zero or one PONV risk factor (68 patients), representing 44% of the analyzed patients (21% risk of PONV), no cases of PONV were observed. In patients with two risk factors (39% risk of PONV), PONV was observed in 3 of 74 patients (4.05%), whereas in patients with three risk factors (61% risk of PONV), it was observed in 3 of 11 patients (27%). On the one hand, liberal PONV prophylaxis is currently recommended in adult patients and children, which should be used even when risk evaluation has not been performed [5]. On the other hand, in this study, among 142 patients (with zero, one, or two risk factors for PONV), the occurrence of PONV was observed only three times (2.11%), i.e., two and a half times lower than that in the study performed by Reibaldi et al., who observed a 5% incidence of PONV in patients receiving VRS under RA and MAC with antiemetic prophylaxis using dexamethasone and ondansetron [1]. Therefore, we hypothesize that the anesthetic regimen based on AoA guidance of GA with IPA using COX-3 inhibitors almost eradicated PONV in low-risk patients. This raises the question of whether following the current guidelines in patients with co-morbidities undergoing VRS would have brought more benefits than harm to these 142 patients in view of possible complications associated with the administration of drugs used for PONV prophylaxis, such as the exemplary QT interval prolongation in healthy individuals, evident even at low ondansetron dosages [72]. A 27% PONV incidence after VRS under AoA-guided GA with IPA using COX-3 inhibitors in patients with at least three risk factors, with a basic 61% risk of PONV, is in concordance with a similar observation made by Feng CD et al., who reported that the SPI guidance for opioid-free anesthesia halved the PONV incidence in patients undergoing thoracoscopic lung resection when compared to opioid-based anesthesia with antiemetic prophylaxis using dexamethasone and ondansetron, despite the group allocation [32]. Further studies in this area are needed on larger groups, including those analyzing a higher percentage of high-risk patients.

In this study, it is hypothesized that the precision of IROA administration using the SPI-guided FNT, along with a stable depth of GA under SE control as AoA guidance, could likely personalize anesthesia for each patient undergoing VRS, in terms of the optimal IROA demand reduced by IPA with intrinsic antiemetic potency and the optimal fluid challenge pertaining to the appropriate depth of volatile anesthesia, an overdose of which is an independent risk factor for PONV.

OCR is a trigeminovagal reflex that is triggered intraoperatively by applying pressure to the eyeball and/or traction of extraocular muscles. Clinically, OCR is defined and, thus, observed as a rapid decrease in heart rate of more than 20% [73]. Its occurrence is a significant intraoperative adverse event, as it can inevitably lead to even serious cardiac arrhythmias, resulting in hemodynamic instability (bradycardia, ectopic beats, nodal rhythm, ventricular fibrillation, or asystole), thus significantly influencing further intraoperative management. According to the current literature, the incidence of OCR is estimated to be 7–70%, depending on the type of ophthalmic surgery and the anesthetic regimen [16,74]. Ghali et al. reported an OCR incidence rate of 27% in patients receiving GA for VRS [16]. In turn, Abdeldayem et al. [75] achieved a 50% decrease in the incidence of OCR by adding sub-Tenon’s block using levobupivacaine for GA in patients undergoing retinal surgery, compared with GA alone. In the current study, OCR was observed in 10% of cases despite group assignment, demonstrating no statistically significant differences between groups. Therefore, the type of IPA has little impact on the incidence of OCR, as observed in our previous project, on which this study was based, and the incidence of OCR was 12% regardless of the group assignment.

In our opinion, the perioperative effects observed in our study can probably also be explained by the employment of SPI-guided IROA titration during AoA-guided GA. The precision of IROA administration, only in the case of ineffective intraoperative analgesia, defined as ∆SPI >15, likely led to stable serum FNT concentration, as changes in the SPI values have been shown to correspond to serum opioid concentrations [26] and have supposedly resulted in the effective suppression of afferent nociceptive stimulation responsible for inducing IPPP. Similarly, we hypothesized that AoA guidance resulted in stable intraoperative analgesia achieved by IROA administration titrated under the observance of SPI values, along with a stable depth of anesthesia reflecting limbic suppression monitored by SE, which attenuated potentially induced OCR by surgical manipulation during the stage of indentation in patients undergoing VRS, as we also demonstrated in our previous analysis of SPI value observations during the detailed stages of AoA-guided GA for VRS [76]. The SPI-guided IROA dosing during GA suppressed afferent nociceptive stimulation evoked by intraoperative muscle traction, which could also prevent central hyperexcitability [21], reducing the OCR rate to a rate close to the lowest rates observed in the literature.

OER was described by Van den Berg et al. as an afferent pathway sharing its limb of the reflex arc with OCR [77] produced by squint surgery in children. Their hypothesis that there is an association between the occurrence of OCR and PONV, particularly early PONV [78], was not proven, as in no case did intraoperative OCR cause PONV in the current study, as well as in our previous project [34]. In further studies, again in our view, the AoA guidance, expressed by stable SE guidance for inhalational anesthetic agent administration and the SPI guidance for IROA titration, may lead to personalized GA and, therefore, result in low rates of OCR and PONV, without any evidence of OER. Similarly, the PONV incidence was not observed to be correlated with the IPPP incidence in this study, although a relationship between IPPP and PONV has been reported in the current literature [54].

This study has several limitations. First, we were unable to examine late PONV in all patients, usually assessed up to 48 h after emergence from GA, because up to half of the patients were enrolled in this study during the COVID-19 pandemic period between lockdowns, and they were discharged from the DO as soon as the effects of anesthetics had subsided. Nevertheless, the incidence of late PONV may have little influence on the final results of overall PONV calculated as early PONV in the PACU and late PONV in the DO, as in our previous project, and no late PONV was observed in patients receiving intravenous preventive analgesia; only cases of late PONV were observed in patients receiving regional analgesia techniques at a similar rate to our current observations.

The presence of IPPP is a subjective phenomenon, the quantification of which is burdensome [79]. This study did not include a control group without AoA guidance on purpose, as studies with such control groups have already been conducted and their results are widely available. Moreover, the digital monitoring of nociception/anti-nociception balance has become a standard anesthetic modality in many centers; thus, obtaining consent for the study from a Bioethical Committee in view of strict regulations, requiring potential benefits from participation in the study, despite the group allocation, may no longer be possible. We also did not deliberately analyze the IPPP incidence after discharge from the recovery room to the DO, because this study included the monitoring of NPRS and SPI at Stage 5, while patient arousal (changing position in bed, cough, etc.) has been shown to significantly interfere with the SPI monitoring [80], thus making such a comparison neither clinically nor scientifically meaningful. Finally, a single dose of intravenous metamizole or paracetamol is known to have an effect for up to several hours; thus, the comparison of NPRS values over a period longer than four hours was found to be of little clinical relevance [81]. All patients received oral midazolam preoperatively, which has been shown to have antiemetic properties in patients undergoing VRS [82], which may have proportionally influenced the final total PONV incidence and the final outcomes across all groups.

In summary, in this study, we observed no advantage of combining IPA with 1 g of paracetamol and 2.5 g of metamizole before VRS in terms of the incidence of OCR and PONV compared to their use alone and to the findings from previous studies in which we observed that a single dose of IPA with any COX-3 inhibitor overweighed RA and control techniques without affecting the efficacy of preventive analgesia when AoA guidance was used. The stability of personalized GA using AoA guidance for VRS resulted in an overall rate of 4% PONV and 10% OCR, regardless of the group allocation, and the requirement for IROA had no influence on secondary outcomes, including perioperative adverse events.

## 5. Conclusions

We recommend the employment of AoA guidance for GA in patients undergoing VRS with IPA using paracetamol or paracetamol/metamizole to achieve low OCR and PONV incidence, thereby optimizing the demand for IROA as a decent alternative to VRS under RA with MAC for carefully selected patients, especially those with zero, one, or two risk factors of PONV, according to the stratification performed using the Apfel score.

## Figures and Tables

**Table 1:** Patient anthropometric characteristics.

Metrics		Total *N* = 153 (100%)	PM *n* = 52 (34%)	P *n* = 51 (33%)	M *n* = 50 (33%)	*p*-Value
Age X ± SD Me (IQR)	[years]	63.81 ± 11.67 65 (12)	64.44 ± 11.03 67 (11.5)	64.59 ± 11.57 66 (9)	62.58 ± 12.32 64 (14)	NS *p* = 0.7
Gender N (%)	Female	82 (54%)	23 (44%)	35 (69%)	24 (48%)	PM vs. P, *p* = 0.02
	Male	71 (46%)	29 (56%)	16 (31%)	26 (52%)	
Height X ± SD Me (IQR)	[cm]	167.08 ± 9.18 165 (15)	169.51 ± 9.02 167 (12)	163.96 ± 8.28 164 (11)	168.34 ± 9.45 170 (15)	PM vs. P, *p* = 0.02
Weight X ± SD Me (IQR)	[kg]	77.2 ± 15.35 77 (21)	84.97 ± 15.58 82 (27)	73.96 ± 14.78 72.5 (21.5)	74.56 ± 13.92 74.5 (19)	PM vs. P, *p* = 0.004; PM vs. M, *p* = 0.009
BMI X ± SD Me (IQR)	[kg/m^2^]	27.73 ± 4.95 26.72(6.55)	29.55 ± 4.95 27.77(5.89)	27.87 ± 5.3 27.76(7.72)	26.26 ± 4.19 25.6(3.98)	PM vs. M, *p* = 0.007
BMI N (%)	Norm	45 (29%)	8 (15%)	17 (33%)	20 (40%)	NS *p* = 0.2
	Overweight	49 (32%)	14 (27%)	15 (29%)	20 (40%)	
	Obesity	39 (25%)	15 (29%)	15 (29%)	9 (18%)	
HA N (%)	Yes (%)	90 (59%)	24 (46%)	35 (69%)	31 (62%)	PM vs. P, *p* = 0.03
Insulin-dependent DM N (%)	Yes (%)	52 (34%)	16 (31%)	21 (41%)	15 (30%)	NS *p* = 0.4
Insulin-independent DM N (%)	Yes (%)	39 (25%)	12 (23%)	14 (27%)	13 (26%)	NS *p* = 0.9

PM—paracetamol/metamizole; P—paracetamol; M—metamizole; HA—arterial hypertension; DM—diabetes mellitus; SD—standard deviation; IQR—interquartile range; NS—not statistically significant.

**Table 2:** Characteristics of the performed treatments.

Surgery		Total *N* = 153 (100%)	PM *n* = 52 (34%)	P *n* = 51 (33%)	M *n* = 50 (33%)	*p*-Value
Type of surgery N (%)	phacovitrectomy	91 (59%)	29 (56%)	29 (57%)	33 (66%)	NS *p* = 0.5
	pars plana vitrectomy	62 (41%)	23 (44%)	22 (43%)	17 (34%)	
Time of surgery X ± SD Me (IQR)	[min]	47.19 ± 18.91 44 (28)	44.44 ± 19 39 (24)	47.22 ± 18.27 44 (29)	50.02 ± 19.41 51 (31)	NS *p* = 0.3
FNT X ± SD Me (IQR)	[mcg]	149.81 ± 90.81 100 (100)	151.79 ± 79.71 100 (100)	116.28 ± 75.37 100 (100)	180.68 ± 104.11 200 (150)	P vs. M, *p* = 0.004
Intraoperative fluid therapy X ± SD Me (IQR)	[ml]	1042.23 ± 342.61 1000 (450)	1090.38 ± 389.35 1100 (450)	958.7 ± 258.48 1000 (250)	1069 ± 351.08 1000 (450)	NS *p* = 0.05

PM—paracetamol/metamizole; P—paracetamol; M—metamizole; FNT—fentanyl; SD—standard deviation; IQR—interquartile range; NS—not statistically significant.

**Table 3:** Distribution of Apfel scores and patient case histories across study groups.

**Surgery**	**Total** ***N* = 153 (100%)**	**PM** ***n* = 52 (34%)**	**P** ***n*** **= 51 (33%)**	**M** ***n* = 50 (33%)**	***p*** **-Value**
Apfel score X ± SD Me (IQR)	1.56 ± 0.73 2 (1)	1.35 ± 0.76 1 (1)	1.73 ± 0.53 2 (1)	1.6 ± 0.83 2 (1)	*p* = 0.03 (Kruskal–Wallis); NS (Dunn’s post hoc), PM vs. P, *p* = 0.06; PM vs. M, *p* = 0.5; P vs. M, *p* = 1.0
Apfel (%) X ± SD Me (IQR)	32 ± 13 39 (18)	28 ± 12 21 (18)	34 ± 10 39 (18)	33 ± 15 39 (18)	PM vs. P, *p* = 0.04
Motion sickness No/yes N (%)	136 (89%)/17 (11%)	49 (94%)/3 (6%)	47 (92%)/4 (8%)	40 (80%)/10 (20%)	NS *p* = 0.05
History of PONV No/yes N (%)	0 (0%)/153 (100%)	0 (0%)/52 (100%)	0 (0%)/50 (100%)	0 (0%)/51 (100%)	-
Smoking No/yes N (%)	137 (90%)/6 (10%)	43 (83%)/9 (17%)	49 (96%)/2 (4%)	45 (90%)/5 (10%)	NS *p* = 0.08
Postoperative use of opioid drugs No/yes N (%)	153 (100%)/0 (0%)	52 (100%)/0 (0%)	50 (100%)/0 (0%)	51 (100%)/0 (0%)	-

PM—paracetamol/metamizole; P—paracetamol; M—metamizole; PONV—postoperative nausea and vomiting; SD—standard deviation; IQR—interquartile range; NS—not statistically significant.

**Table 4:** Postoperative pain occurrence in the study groups.

Post-Operative Pain		Total *N* = 153 (100%)	PM *n* = 52 (34%)	P *n* = 51 (33%)	M *n* = 50 (33%)	*p*-Value
NPRS max X ± SD Me (IQR)	[0÷10]	0.99 ± 1.81 0 (2)	0.54 ± 1.36 0 (0)	1.12 ± 1.87 0 (2)	1.32 ± 2.07 0 (3)	NS *p* = 0.1
Type of first pain perception N (%)	Mild	137 (90%)	51 (98%)	45 (88%)	41 (82%)	PM vs. M, *p* = 0.02
	Moderate	14 (9%)	0 (0%)	5 (10%)	9 (18%)	
	Acute	2 (1%)	1 (2%)	1 (2%)	0 (0%)	

PM—paracetamol/metamizole; P—paracetamol; M—metamizole; NPRS—numerical rating scale; SD—standard deviation; IQR—interquartile range; NS—not statistically significant.

**Table 5:** Incidence of adverse events after vitrectomy/phacovitrectomy in patients depending on their group allocation.

Surgery	Total *N* = 153 (100%)	PM *n* = 52 (34%)	P *n* = 51 (33%)	M *n* = 50 (33%)	*p*-Value
IPPP (yes/no) (%)	16 (10%)/137 (90%)	1 (2%)/51 (98%)	6 (12%)/45 (88%)	9 (18%)/41 (82%)	PM vs. M, *p* = 0.02
OCR (yes/no) (%)	16 (10%)/137 (90%)	3 (6%)/49 (94%)	6 (12%)/45 (88%)	7 (14%)/43 (86%)	NS *p* = 0.4
Overall PONV (yes/no) (%)	6 (4%)/147 (96%)	3 (6%)/49 (94%)	1 (2%)/50 (98%)	2 (4%)/48 (96%)	NS *p* = 0.8
OER (OCR + PONV) (yes/no) (%)	0 (0%)/153 (100%)	0 (0%)/52 (100%)	0 (0%)/50 (100%)	0 (0%)/51 (100%)	-
IPPP + PONV (yes/no) (%)	0 (0%)/153 (100%)	0 (0%)/52 (100%)	0 (0%)/50 (100%)	0 (0%)/51 (100%)	-
PACU					
PONV in the PACU (yes/no) (%)	6 (4%)/147 (96%)	3 (6%)/49 (94%)	1 (2%)/50 (98%)	2 (4%)/48 (96%)	NS *p* = 0.8
Nausea in the PACU (yes/no) (%)	5 (3%)/148 (97%)	2 (4%)/50 (96%)	1 (2%)/50 (98%)	2 (5%)/48 (96%)	NS *p* = 0.8
Vomiting in the PACU (yes/no) (%)	1 (1%)/152 (99%)	1 (2%)/51 (98%)	0 (0%)/50 (100%)	0 (0%)/51 (100%)	-
DO					
PONV in the DO (yes/no) (%)	2 (1%)/151 (99%)	2 (4%)/50 (96%)	0 (0%)/50 (100%)	0 (0%)/51 (100%)	-
Nausea in the DO (yes/no) (%)	2 (1%)/151 (99%)	2 (4%)/50 (96%)	0 (0%)/50 (100%)	0 (0%)/51 (100%)	-
Vomiting in the DO (yes/no) (%)	0 (0%)/153 (100%)	0 (0%)/52 (100%)	0 (0%)/50 (100%)	0 (0%)/51 (100%)	-
PONV in PACU and DO					
PONV in PACU and DO (yes/no) (%)	2 (1%)/151 (99%)	2 (4%)/50 (96%)	0 (0%)/50 (100%)	0 (0%)/51 (100%)	-
Nausea in PACU and DO (yes/no) (%)	2 (1%)/151 (99%)	2 (4%)/50 (96%)	0 (0%)/50 (100%)	0 (0%)/51 (100%)	-
Vomiting in PACU and DO (yes/no) (%)	0 (0%)/153 (100%)	0 (0%)/52 (100%)	0 (0%)/50 (100%)	0 (0%)/51 (100%)	-

PM—paracetamol/metamizole; P—paracetamol; M—metamizole; PONV—postoperative nausea and vomiting; IPPP, intolerable postoperative pain perception; OCR—oculocardiac reflex; OER—oculoemetic reflex; PACU—postoperative care unit; DO—Department of Ophthalmology; NS—not statistically significant.

**Table 6:** Apfel score of patients depending on the presence or absence of PONV in the postoperative period.

Apfel Score [Point] *n* (%)	0 (10% Risk of PONV)	1 (21% Risk of PONV)	2 (39% Risk of PONV)	3 (61% Risk of PONV)	*p*-Value
Total *N* = 153 (100%)	11 (7%)	57 (37%)	74 (48%)	11 (7%)	0 vs. 1, *p* < 0.001; 0 vs. 2, *p* < 0.001; 1 vs. 3, *p* < 0.001; 1 vs. 3, *p* < 0.001
PONV *n* = 6 (4%)	0 (0%)	0 (0%)	3 (50%)	3 (50%)	3 vs. 1, *p* = 0.02
No-PONV *n* = 147 (96%)	11 (7%)	57 (37%)	71 (48%)	8 (5%)	

PONV—postoperative nausea and vomiting.

## Data Availability

The data used to support the findings of this study are included within the article.

## Abbreviations

The following abbreviations are used in this manuscript:

AoA	adequacy of anesthesia
COX-3	cyclooxygenase-3
DM	diabetes mellitus
DO	Department of Ophthalmology
ECG	Electrocardiography
etCO2	end-tidal carbon dioxide
FeAA	fraction of expired sevoflurane
FiAA	fraction of inspired sevoflurane
FiO2	fraction of inspired oxygen
GA	general anesthesia
HA	arterial hypertension
HR	heart rate
IPA	intravenous preventive analgesia
IPPP	intolerable postoperative pain perception
IROA	intravenous rescue opioid analgesia
M	metamizole
MAC	monitored anesthesia care
NIBP	non-invasive blood pressure
NPRS	numeric pain rating scale
OCR	oculocardiac reflex
OER	oculoemetic reflex
P	paracetamol
PACU	postoperative care unit
PM	paracetamol/metamizole
PONV	perioperative nausea and vomiting
RA	regional anesthesia
RE	response entropy
SaO2	arterial oxygen saturation
SE	state entropy
SPI	surgical pleth index
VRS	vitreoretinal surgery
